# Nuclear ErbB2 expression in hepatocytes in liver disease

**DOI:** 10.1007/s00428-020-02871-z

**Published:** 2020-06-26

**Authors:** Paula Döring, Diego F. Calvisi, Frank Dombrowski

**Affiliations:** 1 Institute of Pathology, Universitätsmedizin Greifswald, Friedrich-Loeffler-Straße 23e, 17475 Greifswald, Germany; 2 Institute of Pathology, University of Regensburg, Regensburg, Germany

**Keywords:** Alcoholic steatohepatitis, Fatty liver disease, Liver cirrhosis, ErbB2, EGFR

## Abstract

ErbB2 is a prominent representative of the epidermal growth factor receptors that mainly attract attention as oncogenic drivers and therapeutic targets in cancer. Besides transmembrane signaling, ErbB2 may also translocate into the nucleus and mediate distinct nuclear signaling effects including DNA repair and cell cycle arrest. Unexpectedly, we found nuclear ErbB2 expression in human hepatocytes in various liver diseases so we aimed to investigate the characteristics of liver disease leading to nuclear ErbB2 translocation. The immunohistochemical pattern of ErbB2 staining was analyzed in 1125 liver biopsy samples from patients with hepatic dysfunction. Further signaling and metabolic markers were analyzed by immunohistochemistry in selected liver biopsy samples. We found a cytoplasmic and nuclear ErbB2 expression in hepatocytes from different disease conditions with the strongest expression detected in alcoholic steatohepatitis. Nuclear ErbB2 positivity significantly correlated with histologic parameters of hepatocellular damage including inflammatory activity in steatohepatitis, hepatocellular ballooning, and cholestasis. ErbB2 overexpressing hepatocytes revealed an increase of phospho-STAT3, a downstream effector of nuclear ErbB2 signaling. Notably, we observed in nuclear ErbB2-positive hepatocytes a downregulation of estrogen receptor expression. In alcoholic steatohepatitis and other toxic liver diseases, hepatocytes revealed a nuclear ErbB2 expression implying a so far unknown mechanism in hepatocytes upon cellular stress that might lead to resistance to cell death. Nuclear ErbB2-positive hepatocytes showed downregulation of estrogen receptor expression and increased levels of pSTAT3, which are signs of functionality of nuclear ErbB2 signaling. Furthermore, analysis of hepatocellular ErbB2 expression could serve as helpful tool for diagnosis of liver disease.

**Electronic supplementary material:**

The online version of this article (10.1007/s00428-020-02871-z) contains supplementary material, which is available to authorized users.

## Introduction

ErbB2 is one of the Epidermal Growth Factor Receptors (EGFR), a group of transmembrane receptors with tyrosine kinase activity that are expressed in many tissues and mediate signals of proliferation and cell growth vitally important for organ development and tissue regeneration. Four subtypes of EGF receptors have been identified: EGFR I–IV, synonymously designated as ErbB (avian viral erythroblastosis oncogene) 1–4 or HER1–4.

Common ligands of EGFRs are TGFα, amphiregulin, neuregulins, and others [12]. For ErbB2 (synonymous EGFR II or HER2/neu), no ligand is known; however, ErbB2 heterodimerizes with the other EGFR subtypes resulting in broad and overlapping effects of ErbB2 signaling.

Upon ErbB2 activation, multiple intracellular signaling cascades are involved like the mitogen-activated protein kinase (MAPK), the phosphatidylinositol 3-kinase (PI3K)-AKT pathways, signal transducer and activator of transcription proteins (STAT), SRC tyrosine kinase, and mammalian target of rapamycin (mTOR) [34] mediating proliferation and pro-survival effects.

Unsurprisingly, mutations in these receptors leading to constitutive activation represent common oncogenic driver mutations and therefore particularly EGFR I and ErbB2 are two of the most frequently investigated receptors in tumorigenesis.

In case of ErbB2 receptor, overexpression is frequently caused by gene amplification as observed in breast cancer and gastric cancer [8]. ErbB2 expression status is typically evaluated by immunohistochemistry. Strong circular membranous expression of ErbB2 is stated as ErbB2 positive. Cytoplasmic and/or nuclear staining is very rare (observed in gastric cancer [31]) and is not considered for diagnostic determination of ErbB2 overexpression.

Intensive research on EGFR I and ErbB2 in tumor cells has led to successful therapeutic strategies targeting these receptors: Anti-EGFR molecules like cetuximab or gefitinib are used in colorectal cancer, lung cancer, head and neck carcinoma, and others. Anti-ErbB2 molecules (for example trastuzumab) are approved for therapy of ErbB2 overexpressing breast cancer or gastric cancer.

For some years now, it has been known that EGFRs can translocate to the nucleus and mediate particular signaling functions: (1) Transcriptional regulation of cyclinD1, iNOS, Aurora-A, COX-2 promoters, and others via a transactivation domain and (2) alteration of p21, p53, PCNA activity, and others via a protein kinase domain [14, 29, 33]. This is shown for proliferating tissue (gravid uterus, embryonal tissue, epithelial cells [14]) as well as for several carcinoma types (oral cancer [14], breast cancer [16]). Nuclear ErbB2 expression in tumor cells results in a poor prognosis and might mediate chemoresistance and radioresistance [3].

Little data are available about (nuclear) EGFR/ErbB2 expression in liver tissue. Marti et al. showed nuclear accumulation of EGFR during rat liver regeneration [18, 19].

In hepatocellular carcinoma (HCC), ErbB2 overexpression is uncommon [10, 32]. Hung et al. showed that in Hep3Bx cells, a hepatitis B virus–triggered HCC cell line, ErbB2 expression mediates migration ability but has no impact on proliferation [11].

In developed countries, one of the most important risk factors for liver cirrhosis and HCC is alcoholic steatohepatitis (ASH) [20]. ASH is histologically characterized by steatosis, hepatocellular ballooning, necroinflammation, and occurrence of Mallory-Denk bodies. ASH comes along with severe hepatocellular damage gradually leading to liver fibrosis, cirrhosis, and development of HCC.

Referring to an incidental finding of hepatocellular ErbB2 expression in liver disease, we characterized ErbB2 expression in human non-tumorous and tumorous liver diseases.

## Methods

### Human liver tissue specimens

A total of 1125 liver core needle biopsies were analyzed in routine diagnostics. The following clinical parameters were available: patient’s age, sex, history of alcohol consumption, transaminases, bilirubin, tests for viral hepatitis, focal liver lesions. Tissue was fixed in 4% neutral buffered formalin, dehydrated, and embedded in paraffin. Two-micrometer thick slides were stained with hematoxylin and eosin (H&E), periodic acid Schiff’s stain (PAS), Prussian blue stain (for iron detection), and Picrosirius red stain (for fibrosis). Comparative histological evaluation was also performed with a subset of liver resection specimens and other tissues from routine diagnostics (gastrointestinal, skin, heart biopsies; renal cell cancer). Furthermore, more than 1200 breast cancer cases were tested for ErbB2 expression in routine diagnostics with the anti-ErbB2-antibody clone 4B5 (Roche Ventana). Institutional Review Board was obtained at the local ethical committee of the Universitätsmedizin Greifswald.

The following diagnostic groups were chosen: No findings (*n* = 38): neither inflammation nor necrosis, steatosis, fibrosis, cholestasis, pathological iron storage, or tumor. Alcoholic steatohepatitis (ASH; *n* = 85) and non-alcoholic steatohepatitis (NASH; *n* = 13) were diagnosed and graded according to the Brunt system [4]. In 72 cases, steatohepatitis exhibited mixed features and could not definitely classified as ASH or NASH, termed as unclear steatohepatitis. Further diagnostic groups were viral hepatitis (*n* = 33), autoimmune hepatitis/ primary biliary cirrhosis (*n* = 23), necrosis (*n* = 26), cholestasis (*n* = 27), HCC (*n* = 74), or liver metastasis (*n* = 312). Fibrosis/cirrhosis (*n* = 60) and steatosis (*n* = 102) of liver tissue were only stated as major diagnosis if no other major finding (inflammation, cholestasis etc.) was present. Further diagnostic cases like other hepatocellular tumors than HCC (*n* = 18), non-hepatocellular liver tumors (*n* = 1), rare liver diseases like storage disease (*n* = 5), and miscellaneous findings like scars, chronic abscess, hematoma, or venous congestion (*n* = 16) were analyzed for ErbB2 expression, too, but not included in statistical analysis due to the smaller number of cases. Finally, for statistical analysis, 865 cases were included.

In the investigated population of north-eastern Germany, liver cirrhosis and HCC most frequently are induced by ethanol consumption. Viral hepatitis (hepatitis A–E virus) is rare in this population (*n* = 33).

The following criteria were graduated for each biopsy: fibrosis (0–6, according to Ishak [13]), chicken wire fibrosis (grades 0 to 3), steatosis (grades 0 to 3 [1]), ballooning of hepatocytes (1, present or 0, not present), Mallory-Denk-bodies (1, present or 0, not present), and inflammatory activity of steatohepatitis (grades 0 to 3 [4]).

### Immunohistochemistry

All liver biopsies were tested for ErbB2 expression by immunohistochemistry with anti-ErbB2-antibody clone 4B5 (Roche Ventana) using the FDA-approved staining kit and the fully automated Ventana Bench Mark staining platform following the manufacturer’s protocol for mammary core needle biopsies. The following alternative primary antibodies were used: clone CB11 (DCS diagnostics), clone SP3 (ThermoFisher Scientific), and the polyclonal antibodies HercepTest (Dako) and ErbB2 (Cell Signaling). For further immunohistochemical analyses, the following antibodies were used: EGFR (clone 5B7, Roche Ventana), ErbB4 (polyclonal, Spring Bioscience), Ki67 (clone MIB-1, Dako), estrogen receptor (clone SP1, Roche Ventana), and phospho-STAT3 (Tyr705, Cell Signaling). All used primary antibodies are listed in supplementary material. Immunohistochemical reactions with primary antibodies, peroxidase-conjugated secondary antibody, and diaminobenzidine substrate were performed on automated immunohistochemistry stainers: BOND Max (Leica, Wetzlar, Germany) or Bench Mark Ultra (Roche Ventana, Oro Valley, Arizona, US). ErbB2 in situ-hybridization was performed using the ZytoDot© 2C SPEC ErbB2/centromere 17 probe (Zytovision, Bremerhaven, Germany) according to the instructions of the manufacturer.

### Scoring of ErbB2 staining

Membranous and/or cytoplasmic and/or nuclear staining of hepatocytes was regarded as positive. ErbB2 grade was evaluated on the basis of the proportion of ErbB2-positive hepatocytes referred to all vital hepatocytes of the liver biopsy: grade 0, less than 1%; grade 1, 1–5%; grade 2, > 5–20%; grade 3, more than 20% positive hepatocytes.

### Statistical analysis

ErbB2 expression scores were evaluated with Student’s *t* test. Test results were considered significant if *p* ≤ 0.05.

## Results

### Patterns of hepatocellular ErbB2 expression

**Fig. 1:**
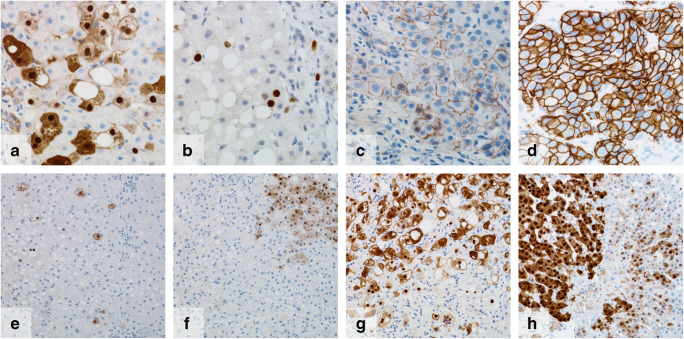
Different ErbB2 expression patterns in hepatocytes. Most commonly, nuclear and cytoplasmic expression (**a**), solely nuclear expression (**b**) or, rarely, solely membranous expression (**c**). Hepatocellular ErbB2 expression clearly differed from membranous expression without nuclear or cytoplasmic colocalization in breast cancer cells (**d**). Varying density of ErbB2 expressing hepatocytes ranging from grades 1 (**e**), 2 (**f**) to 3 (**g**) expression. Variable staining intensity within one sample (**h**, checkered pattern). Length of lower image border **a**–**d**, 0.23 mm; **e**–**h**, 0.47 mm

ErbB2 overexpression is mainly known from different carcinomas like breast cancer or gastric cancer. In these tumors, protein overexpression is caused by *ErbB2* gene amplification and receptor expression is strictly membranous. We analyzed human liver biopsy samples for ErbB2 positivity in immunohistochemistry and observed ErbB2 overexpression in non-neoplastic hepatocytes with different expression patterns (Fig. 1): When hepatocellular ErbB2 expression was present, staining was most frequently strongly nuclear and more or less intensely cytoplasmic (Fig. 1a). Less frequently, the cytoplasmic colocalization was lacking and ErbB2 expression was restricted to the nucleus (Fig. 1b). Rarely, we also observed an exclusively membranous expression without or with only very weak cytoplasmic or nuclear positivity for ErbB2 (Fig. 1c). However, this overall mainly nuclear ErbB2 expression pattern was considerably different from the solely membranous expression that is known from breast cancer cells (Fig. 1d).

A strong circular membranous expression like in breast cancer was never observed in hepatocytes of any liver disease. Vice versa, in breast cancer (more than 1200 cases analyzed), we never observed a nuclear expression of ErbB2.

There was no ErbB2 expression in non-parenchymal cells of the liver, i.e., Kupffer cells, quiescent or activated stellate cells, endothelial cells, and lymphocytes. Bile duct epithelial cells sometimes were ErbB2 positive, either membranous or weakly nuclear, but this was not obviously correlated with the hepatocellular positivity and was not focused in this study.

Density of ErB2 expressing hepatocytes varied from single spotted, weakly stained cells to small groups of ErbB2-positive hepatocytes to diffuse extensive expression by many hepatocytes and with high staining intensity. Due to this wide range of expression density, we classified hepatocellular ErbB2 expression in three expression levels (grades 1–3) (Fig. 1e–g). For this grading, nuclear and/or cytoplasmic and/or membranous ErbB2 positivity was included.

In some cases, staining intensity varied with highly ErbB2 expressing hepatocytes side by side to an area of weakly ErbB2-positive hepatocytes resulting in a checkered pattern (Fig. 1h).

ErbB2 staining results were comparable using three different ErbB2 antibodies targeting the intracellular domain of ErbB2 (clone 4B5, CB11; Herceptest©). With the one ErbB2 antibody clone recognizing the extracellular receptor domain of ErbB2 (clone SP3), hepatocellular staining was negative.

In hepatocytes with nuclear, cytoplasmic, or membranous ErbB2 positivity, we did not observe an amplification of the *ErbB2* gene by in situ hybridization (suppl. Fig. 1).

### ErbB2 expression in liver disease

**Fig. 2:**
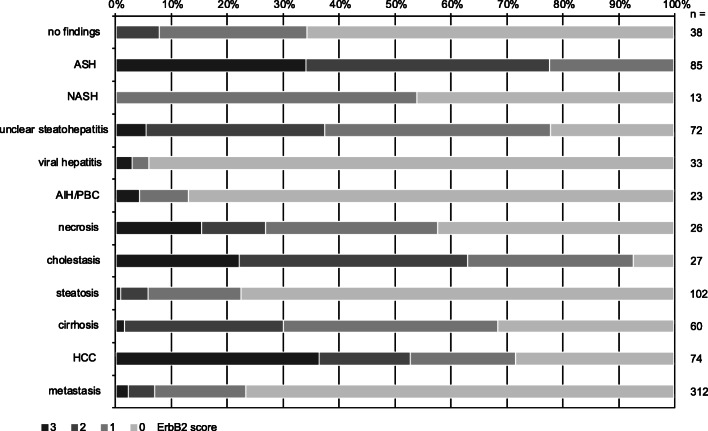
Proportion of ErbB2-positive cases dependent on diagnosis of liver disease. The highest frequency of hepatic ErbB2 expression and also the highest density of ErbB2 expressing hepatocytes were found in alcoholic steatohepatitis (ASH). Other hepatitides as well as non-alcoholic steatohepatitis (NASH) and steatosis without concomitant inflammation were mainly ErbB2 negative. A strong association to ErbB2 expression was also observed in cholestatic liver diseases, in subacute confluent hepatocelluar necrosis, and in hepatocellular carcinoma (ErbB2-positive tumor cells). In metastatic liver disease, hepatocytes sometimes were ErbB2 positive

**Fig. 3:**
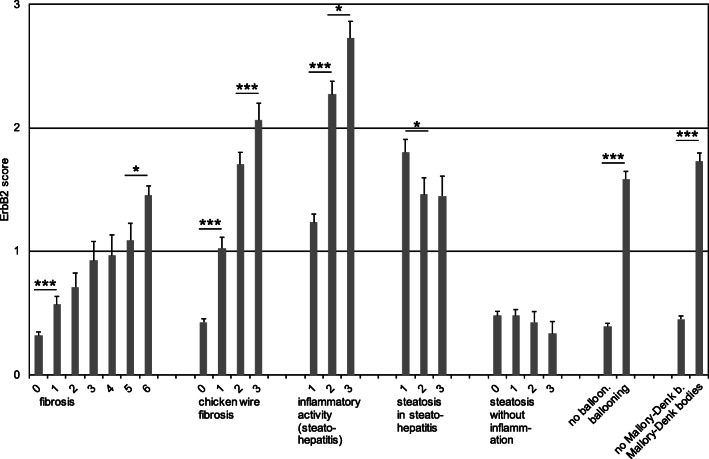
Hepatocellular ErbB2 positivity correlated with distinct histomorphologic features of liver injury. ErbB2 scores of liver tissue samples correlated with progressive liver fibrosis, particularly chicken wire fibrosis, irrespective of the presence of inflammation. Severity of steatohepatitis (inflammatory activity) and particularly hepatocellular ballooning and presence of Mallory-Denk-bodies significantly correlated with ErbB2 positivity. In case of steatosis with or without concomitant steatohepatitis, ErbB2 scores did not correlate with grade of steatosis. Depicted are means of ErbB2 scores with SEM. *p ≤ 0.05, ***p ≤ 0.0001

Eight hundred sixty-five liver biopsy samples obtained due to hepatic dysfunction or hepatic foci were scored for nuclear, cytoplasmic, or membranous ErbB2 expression levels 0–3 as explained above. Figure 2 demonstrates the distribution of ErbB2 expression levels dependent on leading diagnosis. The strongest association of ErbB2-positive cases was observed with diagnosis of alcoholic steatohepatitis (ASH). All liver biopsies with diagnosis of ASH showed an at least weak (score 1), mainly nuclear localized expression of ErbB2, more than 30% even a strong expression (score 3). Scores of hepatocellular ErbB2 expression were significantly correlated with inflammatory activity of ASH. Nuclear ErbB2 positivity was particularly strong in ballooned hepatocytes (Fig. 3).

Strikingly, in non-alcoholic steatohepatitis (NASH), ErbB2 expression was low (score 1) to negative and reached in no case a medium (score 2) to strong (score 3) expression level. ErbB2 scores in ASH were significantly higher than in NASH even if the groups NASH and unclear steatohepatitis were pooled for statistical analysis. In general, ErbB2 expression significantly correlated with inflammatory activity of steatohepatitis (ASH and NASH) (Fig. 3).

In other hepatitides, i.e., viral hepatitis, autoimmune hepatitis, drug-induced hepatitis, or graft-versus-host disease, ErbB2 immunoreactivity of liver tissue was negative in most cases independent of inflammatory activity. The few cases with moderate or strong ErbB2 expression additionally showed a moderate or severe steatosis.

Furthermore, hepatic steatosis by itself without concomitant steatohepatitis presented in most cases as ErbB2 negative, too (suppl. Fig. 2), and there were no differences in ErbB2 scores between different grades of steatosis (Fig. 3). However, in steatohepatitis, there was even a significant inverse correlation between degree of steatosis and mean score of ErbB2 (Fig. 3).

Other liver diseases associated with a common ErbB2 expression were cholestatic hepatopathies and cases with subacute confluent hepatocellular necrosis following toxic injury (Fig. 2), severe hepatocellular siderosis, and Wilson’s disease. Of note, in cholestasis, ErbB2 expression of hepatocytes was often confined to the cell membranes without additional cytoplasmic or nuclear expression (Fig. 1c and suppl. Fig. 3). In cholestatic hepatopathy and also some other liver diseases, ballooning of hepatocytes, as mentioned above in case of ASH, can be observed. Strikingly, ballooning was clearly associated with ErbB2 positivity of these cells (Fig. 3). As ballooned hepatocytes often contained Mallory-Denk bodies, the finding of Mallory-Denk bodies was also significantly associated with ErbB2-positive hepatocytes (Fig. 3). No liver sample containing Mallory-Denk bodies was ErbB2 negative. Fibrosis score as well as score of chicken wire fibrosis correlated significantly with increasing scores of ErbB2 expression (Fig. 3).

**Fig. 4:**
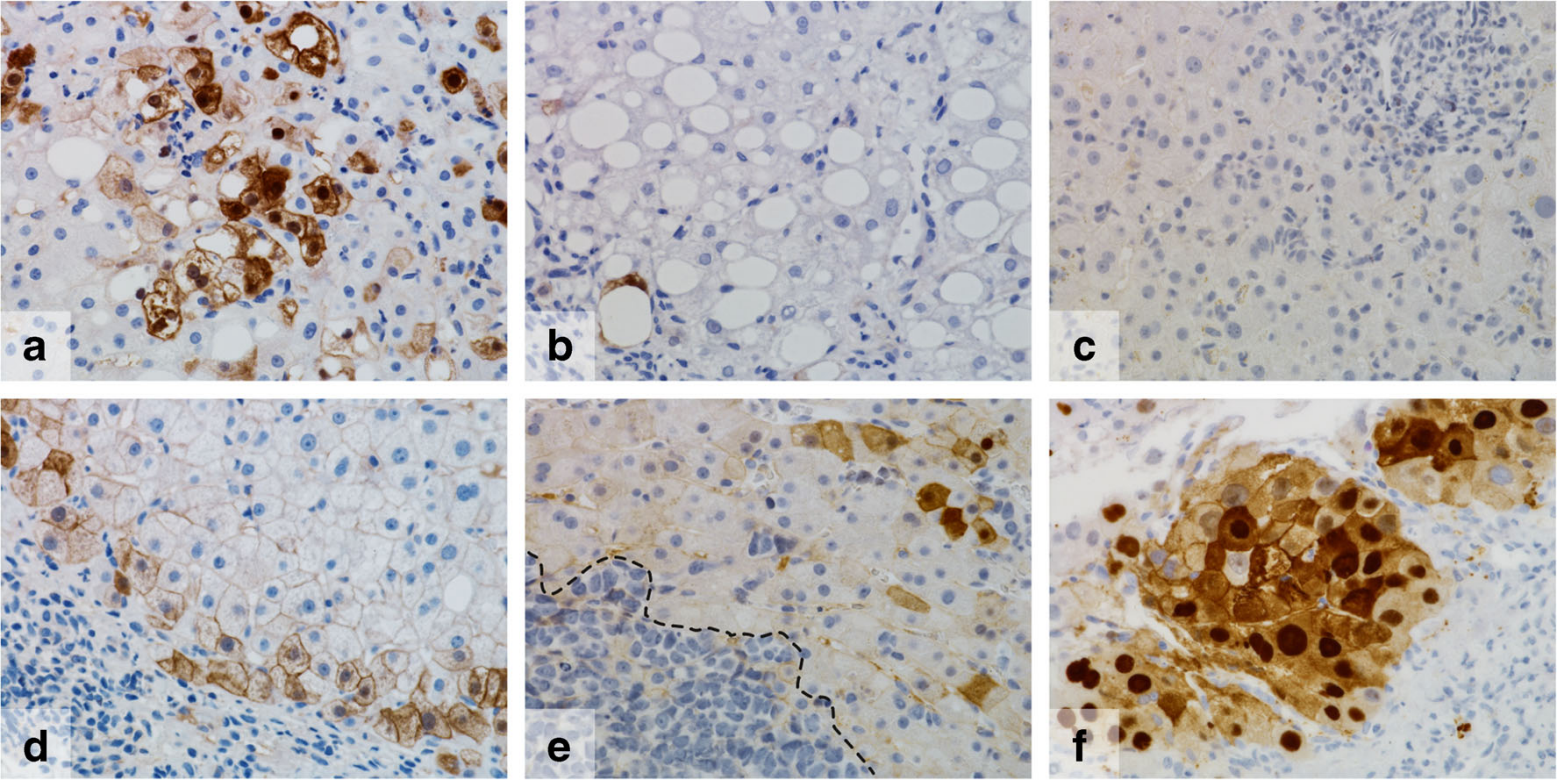
ErbB2 expression in different liver diseases. Groups of ErbB2-positive hepatocytes in ASH (**a**). Only single positive cells in NASH (**b**) and completely ErbB2-negative liver tissue in viral hepatitis type E (**c**). Gradient of ErbB2-positive hepatocytes in cryptogenic cirrhosis to the periphery of a cirrhosis nodule (**d**). Small groups of ErbB2-positive hepatocytes in proximity to a liver metastasis (in this case small cell lung cancer, lower left corner) (**e**). Strongly positive tumor cells of HCC (**f**). Length of lower image border **a**–**f**, 0.31 mm

Liver cirrhosis, as result of different disease conditions like ASH, revealed a mixed presentation of ErbB2 expression levels. ErbB2 expression in cirrhosis often was confined to the hepatocellular layers in the periphery of the cirrhosis nodules (Fig. 4) resulting in a gradient of ErbB2 expression within the cirrhosis nodule.

Interestingly, tumor cells of more than 50% of HCC cases showed an ErbB2 expression and expression levels were in case of ErbB2 positivity particularly strong (score 3) (in suppl. Fig. 4, an example of only weakly ErbB2-positive HCC is depicted).

Histologically inconspicuous liver tissue generally revealed no or in rare cases a weak ErbB2 expression. Of note, in proximity to metastatic tumor masses, we sometimes observed some spotted ErbB2-positive hepatocytes without another underlying liver disease (Fig. 4).

We checked other tissues sharing the morphologic aspect of ballooning of altered cells, i.e., mucoid degenerated cardiomyocytes after sublethal ischemia, clear cell renal cell carcinoma, and human papilloma virus–associated cell swelling of squamous cells of the skin, esophagus, and cervix uteri for ErbB2 expression with consistently negative findings. ErbB2 positivity of non-neoplastic tissue, also with nuclear expression, we found in bile cholangiocytes and gastric foveolar epithelia in some cases (suppl. Fig. 5).

### Altered expression of growth hormone receptor and steroid hormone receptor in ErbB2-positive liver tissue

**Fig. 5:**
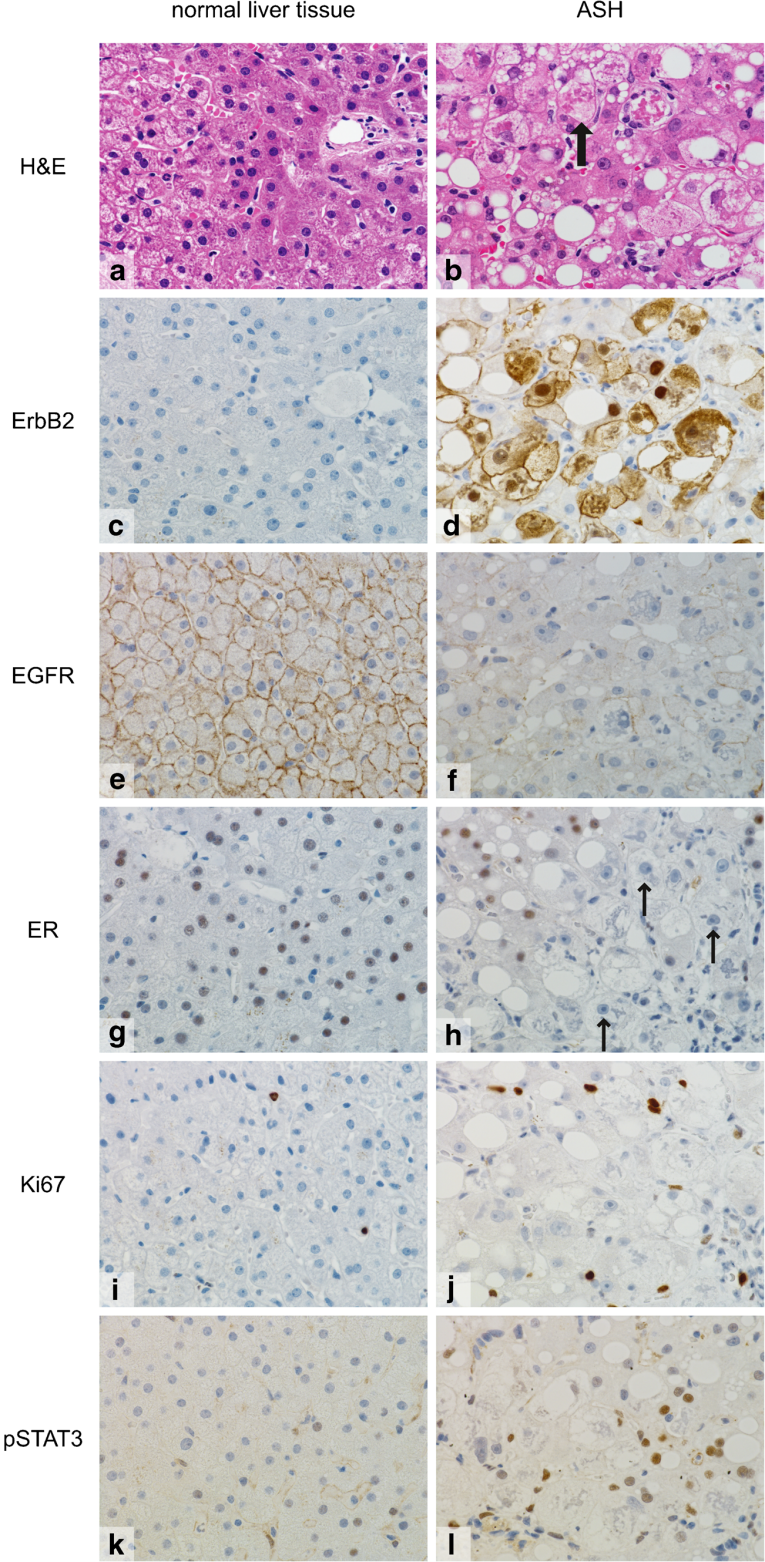
**a**–**l** Additional changes of cellular metabolism in ErbB2-positive hepatocytes in ASH: Left column: Histologically normal liver tissue. Right column: ASH (note ballooned hepatocytes containing Mallory-Denk bodies (thick arrow)). In ErbB2 expressing hepatocytes in ASH, EGFR expression was decreased, estrogen receptor expression clearly downregulated (thin arrows), proliferative activity (Ki67) slightly increased, and phospho-STAT3 expression increased. Length of lower image border **a**–**l**, 0.19 mm

Hepatocellular ErbB2 expression was accompanied by further alterations of hepatocellular growth factor expression: EGFR I, constitutively membranous expressed in hepatocytes, commonly was depressed in liver tissue with a strong ErbB2 positivity (Fig. 5). Of note and in contrast to ErbB2, at no time, we observed a nuclear localization of EGFR I. We did not find a hepatocellular ErbB4 expression neither in ErbB2-positive nor in ErbB2-negative cases. Strikingly, in ErbB2-positive hepatocytes, independent from the disease background, estrogen receptor (ER) expression was considerably downregulated (Fig. 5). Hepatocytes containing Mallory-Denk bodies were ER negative. Most ballooned hepatocytes were ER negative. Commonly, ER downregulation also affected the ErbB2-negative hepatocytes in direct proximity to ErbB2-positive hepatocytes resulting in an even bigger proportion of ER-negative than ErbB2-positive hepatocytes (suppl. Fig. 6).

Constitutive hepatocellular progesterone receptor and androgen receptor expression were not altered in ErbB2-positive liver tissue (tested with 25 cases). Furthermore, no nuclear translocation of beta-catenin was observed.

### Effects of ErbB2 expression

Proliferative activity (Ki67 index) of ErbB2-positive liver tissue commonly was slightly increased compared with histologically normal liver tissue but ErbB2-positive cells themselves did not show an enhanced proliferative activity so increased proliferation could be attributed to reparative effects following inflammatory liver injury. Regenerative and proliferative activity of hepatocytes in viral or autoimmune hepatitis was much higher than in ASH but those cases almost always were ErbB2 negative as described above. Phosphorylated STAT3, a downstream effector of nuclear ErbB2, was increased in ErbB2-positive liver tissue.

## Discussion

Many growth factor receptors, including EGF receptors [5, 22, 23] and fibroblast growth factor (FGF) receptor [5, 17], are usually well known as receptor tyrosine kinases localized at the cell membrane but have been found in the nucleus as well. Their nuclear functions are only partially understood. Nuclear expression of ErbB2 and other EGFRs is mainly found in tumor cells [14, 16] but also in proliferating cells during embryonal development [14].

To the best of our knowledge, this is the first description of nuclear ErbB2 expression in hepatocytes in human liver disease. Most frequently, we detected ErbB2 expression in alcoholic steatohepatitis (ASH). ErbB2 positivity of liver tissue correlated with severity of inflammation and signs of longstanding liver injury like liver fibrosis. By contrast, in non-alcoholic steatohepatitis (NASH), we detected a significantly lower ErbB2 positivity. Steatosis without concomitant steatohepatitis was not associated with nuclear ErbB2 expression, either. Furthermore, ErbB2 expression was not observed in case of immunologic liver inflammation with immune cell–mediated apoptosis of hepatocytes like viral hepatitis and autoimmune hepatitis.

Thus, alcohol seems to be the trigger of hepatocellular ErbB2 expression presumably due to its direct hepatotoxic or indirect lipotoxic properties. This assumption is supported by single cases following exposure to other liver-toxic agents like herbicides that also revealed a strong nuclear ErbB2 expression.

Cholestatic liver disease was a further entity associated with frequent ErbB2 expression in this study. Interestingly, ErbB2 expression pattern was different than described before; in cholestatic liver disease, hepatocytes showed an accentuated membranous ErbB2 expression.

Cellular mechanisms inducing ErbB2 expression in hepatocytes upon alcohol or bile acid/ bile salt exposure might be damage of cell membrane integrity possibly by detergent-like effects or release of reactive oxygen species as a result of cell organelle damage.

In the present study, hints for the functionality of nuclear ErbB2 in hepatocytes are the enhanced expression of phosphorylated STAT3, a downstream effector of nuclear ErbB2 described in previous studies [2, 7, 15], and the downregulation of hepatocellular estrogen receptor (ER) expression.

Initially, this interesting finding of ER downregulation was difficult to integrate in context to ErbB2 expression because the physiologic role of ER in hepatocytes is largely unknown and in some way contradictory. Estrogens seems to support liver cell regeneration [27, 28] but in hepatocellular carcinoma (HCC) also act as protective factor against tumor progression [30], suppress tumor cell proliferation, and promote tumor cell apoptosis [25].

In concordance with our observations, Erkan et al. found a lower ER expression in patients with NASH compared with human liver samples with simple steatosis or normal liver tissue [6].

In breast cancer cells, previous studies showed that ErbB2 itself in shape of its p95 carboxy-terminal fragment [24] or via an ErbB2-encoded miRNA downregulate ER expression [21]; so in breast cancer, ErbB2 amplification is suspected to lead to endocrine therapy resistance.

The effects of nuclear ErbB2 expression in toxic and cholestatic liver injury on hepatocyte fate and disease course are unclear. In this study, we could not observe a direct proliferative effect of nuclear ErbB2 expression in hepatocytes as one would have supposed as likely effect of a growth factor receptor. Possibly, ErbB2 expression mediates survival effects in injured hepatocytes. Interestingly, ballooned hepatocytes, which are often supposed as degenerative, dying cells, revealed a particularly strong ErbB2 expression. This survival signal weakens the previous dogma of dying hepatocytes and supports the hypothesis of ballooned hepatocytes as undead or senescent cells [9].

In various tumor entities, particularly breast cancer and gastric cancer, ErbB2 is overexpressed due to ErbB2 gene amplification. Although ErbB2 overexpression goes along with poor prognosis, it also opens the opportunity to targeted therapy by ErbB2 directing antibodies like trastuzumab. However, for diagnostic testing of ErbB2 overexpression, only membranous expression is considered. For HCC, ErbB2 overexpression is described as extremely rare event [10, 32]. In the present study, nuclear ErbB2 expression was a frequent finding in HCC. A strong membranous ErbB2 expression as described for breast cancer was not observed in HCC (74 cases). Vice versa, in breast cancer, we never detected a nuclear ErbB2 expression. In ErbB2-positive HCC, ErbB2 gene amplification was not present. ErbB2 expression was not associated with grade of tumor differentiation or morphology. In ErbB2-positive HCC, surrounding non-tumorous liver tissue not necessarily revealed ErbB2-positive hepatocytes, either. Thus, it seems unlikely that ErbB2 expression of injured hepatocytes is conserved in the progression to preneoplastic lesions and HCC but as ErbB2 positivity in HCC seems to be a recent event in tumorigenesis. It is unclear which impact nuclear ErbB2 expression in HCC has on tumor behavior and whether nuclear localized ErbB2 could be therapeutically targeted by trastuzumab and other ErbB2-directing molecules. One study by Tatebe et al. demonstrated a synergistic growth-inhibitory effect of trastuzumab and 9-*cis*-retinoic acid in HLF cells, a HCC cell line [26]. However, ErbB2 expression in HLF cells was not evaluated.

In conclusion, we presented a precise characterization of liver disease associated with hepatocellular ErbB2 expression. Even though mechanisms and effects of nuclear ErbB2 expression in hepatocytes so far are not well understood, it is obvious by now that assessment of ErbB2 positivity in liver tissue can serve as useful diagnostic tool. Nuclear ErbB2 expression is a diagnostic marker for toxic injury; membranous ErbB2 positivity indicates cholestatic liver disease. And assessment of ErbB2 expression is helpful in differential diagnosis of HCC and other tumor infiltrates in liver tissue.

Furthermore, this study opens a wide field of research about functional implications of nuclear ErbB2 signaling deducing possible therapeutic strategies in non-neoplastic liver disease and HCC.

## Electronic supplementary material

ESM 1 (DOCX 1401 kb).

## References

[1] Bedossa P, Poitou C, Veyrie N, Bouillot JL, Basdevant A, Paradis V, Tordjman J, Clement K (2012). Histopathological algorithm and scoring system for evaluation of liver lesions in morbidly obese patients. Hepatology.

[2] Béguelin W, Díaz Flaqué MC, Proietti CJ (2010). Progesterone receptor induces ErbB-2 nuclear translocation to promote breast cancer growth via a novel transcriptional effect: ErbB-2 function as a coactivator of Stat3. Mol Cell Biol.

[3] Brand TM, Iida M, Luthar N, Starr MM, Huppert EJ, Wheeler DL (2013). Nuclear EGFR as a molecular target in cancer. Radiother Oncol J.

[4] Brunt EM (2016) Nonalcoholic fatty liver disease: pros and cons of histologic systems of evaluation. Int J Mol Sci 17. 10.3390/ijms17010097 10.3390/ijms17010097 PMC4730339 26771611

[5] Carpenter G (2003). Nuclear localization and possible functions of receptor tyrosine kinases. Curr Opin Cell Biol.

[6] Erkan G, Yilmaz G, Konca Degertekin C, Akyol G, Ozenirler S (2013). Presence and extent of estrogen receptor-alpha expression in patients with simple steatosis and NASH. Pathol Res Pract.

[7] Han W, Carpenter RL, Cao X, Lo H-W (2013). STAT1 gene expression is enhanced by nuclear EGFR and HER2 via cooperation with STAT3. Mol Carcinog.

[8] Hechtman JF, Polydorides AD (2012). HER2/neu gene amplification and protein overexpression in gastric and gastroesophageal junction adenocarcinoma: a review of histopathology, diagnostic testing, and clinical implications. Arch Pathol Lab Med.

[9] Hirsova P, Gores GJ (2015). Ballooned hepatocytes, undead cells, sonic hedgehog, and vitamin E: therapeutic implications for nonalcoholic steatohepatitis. Hepatology.

[10] Hsu C, Huang C-L, Hsu H-C, Lee PH, Wang SJ, Cheng AL (2002). HER-2/neu overexpression is rare in hepatocellular carcinoma and not predictive of anti-HER-2/neu regulation of cell growth and chemosensitivity. Cancer.

[11] Hung C-M, Huang W-C, Pan H-L, Chien PH, Lin CW, Chen LC, Chien YF, Lin CC, Leow KH, Chen WS, Chen JY, Ho CY, Hou PS, Chen YJ (2014). Hepatitis B virus X upregulates HuR protein level to stabilize HER2 expression in hepatocellular carcinoma cells. Biomed Res Int.

[12] Hynes NE, Lane HA (2005). ERBB receptors and cancer: the complexity of targeted inhibitors. Nat Rev Cancer.

[13] Ishak K, Baptista A, Bianchi L, Callea F, de Groote J, Gudat F, Denk H, Desmet V, Korb G, MacSween RNM, Phillips MJ, Portmann BG, Poulsen H, Scheuer PJ, Schmid M, Thaler H (1995). Histological grading and staging of chronic hepatitis. J Hepatol.

[14] Lin SY, Makino K, Xia W, Matin A, Wen Y, Kwong KY, Bourguignon L, Hung MC (2001). Nuclear localization of EGF receptor and its potential new role as a transcription factor. Nat Cell Biol.

[15] Lo H-W, Hsu S-C, Ali-Seyed M, Gunduz M, Xia W, Wei Y, Bartholomeusz G, Shih JY, Hung MC (2005). Nuclear interaction of EGFR and STAT3 in the activation of the iNOS/NO pathway. Cancer Cell.

[16] Lo H-W, Xia W, Wei Y (2005). Novel prognostic value of nuclear epidermal growth factor receptor in breast cancer. Cancer Res.

[17] Maher PA (1996). Nuclear translocation of fibroblast growth factor (FGF) receptors in response to FGF-2. J Cell Biol.

[18] Marti U, Hug M (1995). Acinar and cellular distribution and mRNA expression of the epidermal growth factor receptor are changed during liver regeneration. J Hepatol.

[19] Marti U, Wells A (2000). The nuclear accumulation of a variant epidermal growth factor receptor (EGFR) lacking the transmembrane domain requires coexpression of a full-length EGFR. Mol Cell Biol Res Commun.

[20] Neuman MG, French SW, French BA, Seitz HK, Cohen LB, Mueller S, Osna NA, Kharbanda KK, Seth D, Bautista A, Thompson KJ, McKillop IH, Kirpich IA, McClain CJ, Bataller R, Nanau RM, Voiculescu M, Opris M, Shen H, Tillman B, Li J, Liu H, Thomes PG, Ganesan M, Malnick S (2014). Alcoholic and non-alcoholic steatohepatitis. Exp Mol Pathol.

[21] Newie I, Søkilde R, Persson H, Grabau D, Rego N, Kvist A, von Stedingk K, Axelson H, Borg Å, Vallon-Christersson J, Rovira C (2014). The HER2-encoded miR-4728-3p regulates ESR1 through a non-canonical internal seed interaction. PLoS One.

[22] Ni C-Y, Murphy MP, Golde TE, Carpenter G (2001). γ-Secretase cleavage and nuclear localization of ErbB-4 receptor tyrosine kinase. Science.

[23] Offterdinger M, Schöfer C, Weipoltshammer K, Grunt TW (2002). C-erbB-3: a nuclear protein in mammary epithelial cells. J Cell Biol.

[24] Parra-Palau JL, Pedersen K, Peg V, Scaltriti M, Angelini PD, Escorihuela M, Mancilla S, Sanchez Pla A, Ramon y Cajal S, Baselga J, Arribas J (2010). A major role of p95/611-CTF, a carboxy-terminal fragment of HER2, in the down-modulation of the estrogen receptor in HER2-positive breast cancers. Cancer Res.

[25] Shen M, Shi H (2016). Estradiol and estrogen receptor agonists oppose oncogenic actions of Leptin in HepG2 cells. PLoS One.

[26] Tatebe H, Shimizu M, Shirakami Y, Tsurumi H, Moriwaki H (2008). Synergistic growth inhibition by 9-cis-retinoic acid plus trastuzumab in human hepatocellular carcinoma cells. Clin Cancer Res.

[27] Uebi T, Umeda M, Imai T (2015). Estrogen induces estrogen receptor alpha expression and hepatocyte proliferation in the livers of male mice. Genes Cells.

[28] Umeda M, Hiramoto M, Imai T (2015). Partial hepatectomy induces delayed hepatocyte proliferation and normal liver regeneration in ovariectomized mice. Clin Exp Gastroenterol.

[29] Wang Y-N, Yamaguchi H, Hsu J-M, Hung M-C (2010). Nuclear trafficking of the epidermal growth factor receptor family membrane proteins. Oncogene.

[30] Wei Q, Guo P, Mu K, Zhang Y, Zhao W, Huai W, Qiu Y, Li T, Ma X, Liu Y, Chen X, Han L (2015). Estrogen suppresses hepatocellular carcinoma cells through ERβ-mediated upregulation of the NLRP3 inflammasome. Lab Investig.

[31] Woo CG, Ho WJ, Park YS, Park SR, Ryu MH, Jung HY, Kang YK (2017). A potential pitfall in evaluating HER2 immunohistochemistry for gastric signet ring cell carcinomas. Pathology.

[32] Xian Z-H, Zhang S-H, Cong W-M, Wu WQ, Wu MC (2005). Overexpression/amplification of HER-2/neu is uncommon in hepatocellular carcinoma. J Clin Pathol.

[33] Xie Y, Hung MC (1994). Nuclear localization of p185neu tyrosine kinase and its association with transcriptional transactivation. Biochem Biophys Res Commun.

[34] Yarden Y, Sliwkowski MX (2001). Untangling the ErbB signalling network. Nat Rev Mol Cell Biol.

