# Supplementary material for: Nuclear ErbB2 expression in hepatocytes in liver disease
Source: Virchows Arch. 2020 Jun 26;478(2):309–18. doi: 10.1007/s00428-020-02871-z (PMC7969555; doi:10.1007/s00428-020-02871-z)
Supplement: Supplementary file 1 — (DOCX 1401 kb). [file 428_2020_2871_MOESM1_ESM.docx]

**Supplementary material**

**Title:** Nuclear ErbB2 expression in hepatocytes in liver disease

**Journal:** Virchows Archiv

**Authors:** Paula Döring, Diego F. Calvisi, Frank Dombrowski

**Corresponding author:** Dr. Paula Döring, Institute of Pathology, Friedrich-Loeffler-Straße 23e, 17475 Greifswald, Germany, phone: +49 3834 86 5715, fax: +49 3834 86 5704, mail: [paula.doering@med.uni-greifswald.de](mailto:paula.doering@med.uni-greifswald.de)

**Table of contents:**

| **Content** | **Description** | **Page** |
| --- | --- | --- |
| suppl. Fig. 1 | ErbB2 in situ hybridization in ErbB2 positive HCC | 2 |
| suppl. Fig. 2 | Hepatocellular steatosis, ErbB2 negative | 2 |
| suppl. Fig. 3 | Cholestasis with accentuated membranous ErbB2 expression | 3 |
| suppl. Fig. 4 | HCC with very weak ErbB2 expression | 3 |
| suppl. Fig. 5 | Nuclear ErbB2 expression in gastric foveolar epithelia and cholangiocytes | 4 |
| suppl. Fig. 6 | Loss of ER expression in areas of hepatocellular ErbB2 expression | 4 |
| Table of used primary antibodies |  | 5 |


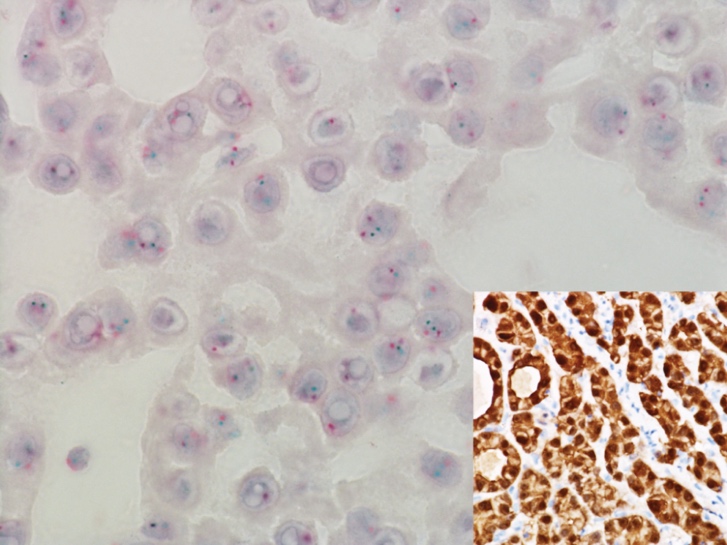


Suppl. Fig. 1: In HCC with strong nuclear ErbB2 expression (see inlay), no ErbB2 gene amplification was observed by in-situ hybridization (green signal: ErbB2 gene; red signal: chromosome 17 centromere). Length of lower image border: 0,14 mm, inlay: 0,23 mm).


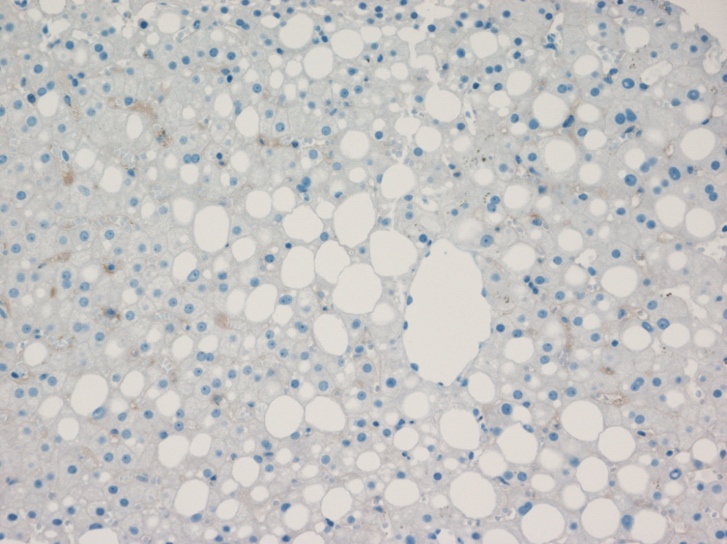


Suppl. Fig. 2: Hepatocellular steatosis without present inflammation usually did not show ErbB2 expression (score 0). Length of lower image border: 0,61 mm.


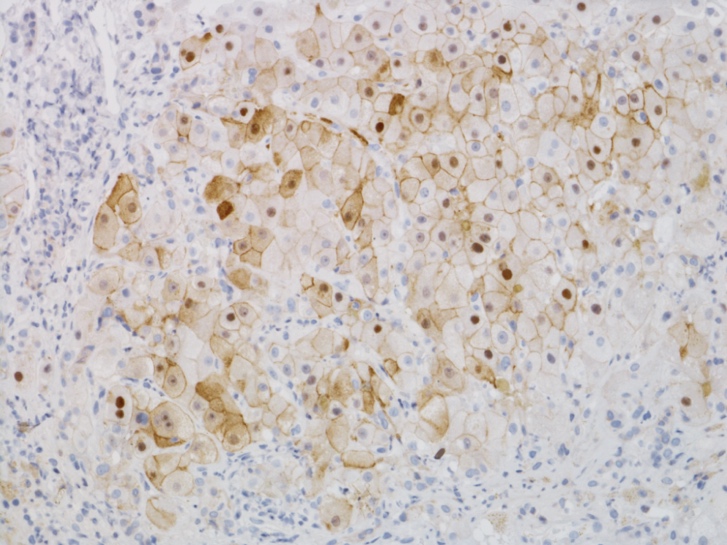


Suppl. Fig. 3: In cholestasis, hepatocytes showed an accentuated membranous ErbB2 expression, with or without concomitant cytoplasmic or nuclear ErbB2 expression. Length of lower image border: 0,62 mm.


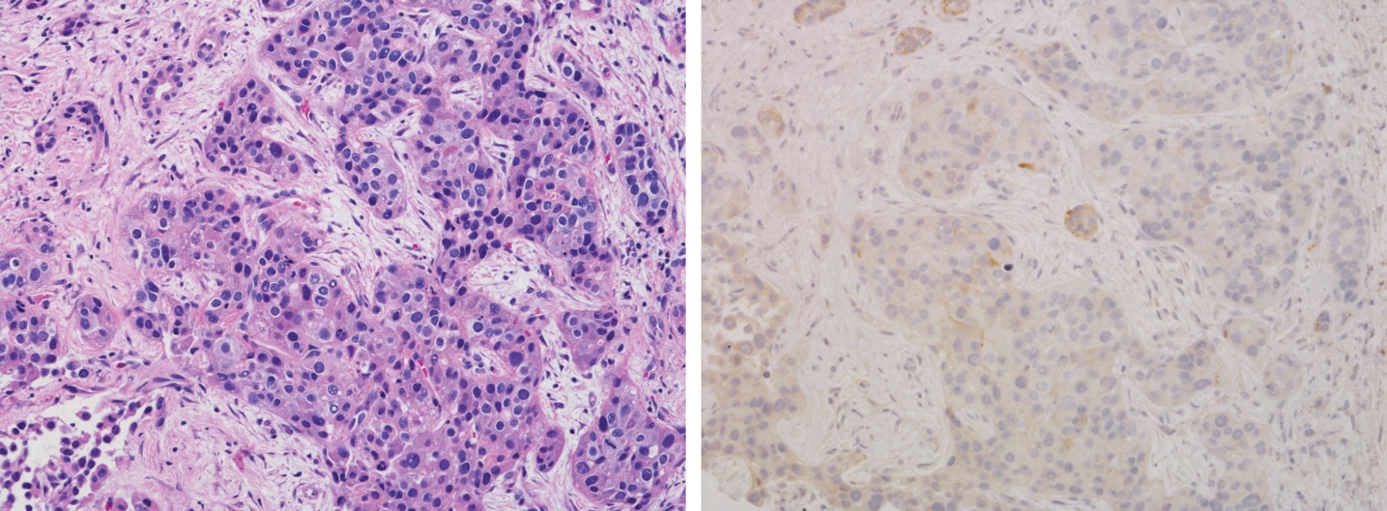


Suppl. Fig. 4: HCC with very weak ErbB2 expression, as it was a rare finding in HCC. Left side: H&E, right side: ErbB2. Length of lower image border: 0,62 mm.


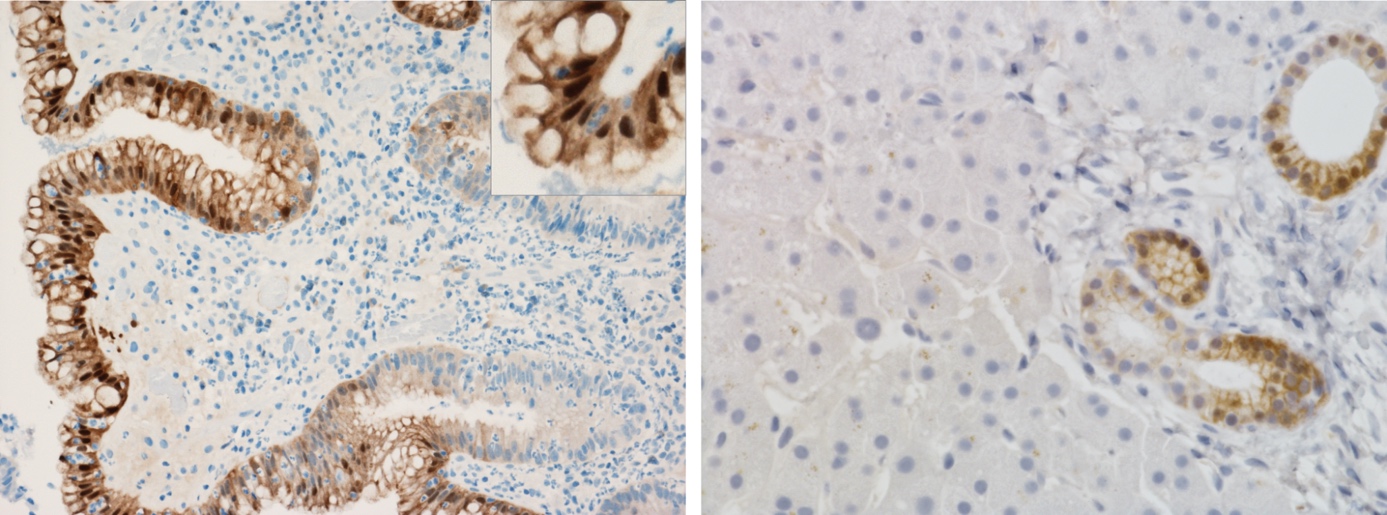


Suppl. Fig. 5: Besides hepatocytes, nuclear ErbB2 expression was frequently observed in non-neoplastic gastric foveolar epithelia (left side) and occasionally in cholangiocytes (right side). Length of lower image border: left image 0,62 mm, right image 0,31 mm.


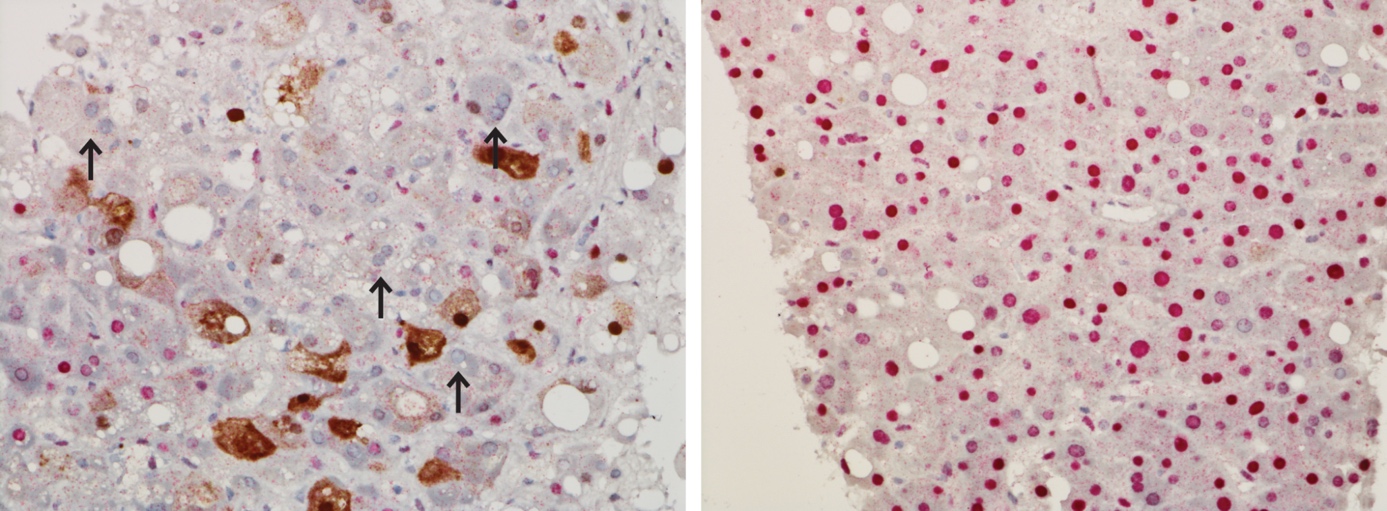


Suppl. Fig. 6: ErbB2 positive hepatocytes (brown staining) and even the neighbouring ErbB2 negative hepatocytes (see arrows) revealed a downregulation of estrogen receptor (ER) expression (red staining). In this Figure a case with alcoholic steatohepatitis is depicted. Left side: Area with ErbB2 positive hepatocytes. Right side: Area with unaffected, ErbB2 negative hepatocytes of the same biopsy; note that ER expression is conserved here. Length of lower image border: 0,41 mm.

| **antibody** | **clone** | **species** | **company** |
| --- | --- | --- | --- |
| androgen receptor | SP107 | rabbit | Ventana Medical Systems, Oro Valley, Arizona, US |
| β-catenin | polyclonal | rabbit | DCS diagnostics, Hamburg, Germany |
| EGFR | 5B7 | rabbit | Ventana Medical Systems, Oro Valley, Arizona, US |
| EGFR sc-03 | polyclonal | rabbit | Santa Cruz, Dallas, Texas, US |
| estrogen receptor | SP1 | rabbit | Ventana Medical Systems, Oro Valley, Arizona, US |
| ErbB2 | 4B5 | rabbit | Ventana Medical Systems, Oro Valley, Arizona, US |
| ErbB2 | CB11 | mouse | DCS diagnostics, Hamburg, Germany |
| ErbB2 | SP3 | rabbit | ThermoFisher Scientific, Waltham, Massachusetts, US |
| ErbB2 | polyclonal | rabbit | Cell Signaling, Danvers, Massachusetts, US |
| ErbB4 | polyclonal | rabbit | Spring Bioscience (Ventana), Pleasanton, California, US |
| Herceptest**©** | polyclonal | rabbit | Dako Agilent, Santa Clara, California, US |
| Ki67 | MIB-1 | mouse | Dako Agilent, Santa Clara, California, US |
| phospho**-**STAT3 | Tyr705 | rabbit | Cell Signaling, Danvers, Massachusetts, US |
| progesteron receptor | 1E2 | rabbit | Ventana Medical Systems, Oro Valley, Arizona, US |
